# Chromatin profiling of cortical neurons identifies individual epigenetic signatures in schizophrenia

**DOI:** 10.1038/s41398-019-0596-1

**Published:** 2019-10-17

**Authors:** Fedor E. Gusev, Denis A. Reshetov, Amanda C. Mitchell, Tatiana V. Andreeva, Aslihan Dincer, Anastasia P. Grigorenko, Gennady Fedonin, Tobias Halene, Maria Aliseychik, Elena Filippova, Zhiping Weng, Schahram Akbarian, Evgeny I. Rogaev

**Affiliations:** 1 Department of Psychiatry, University of Massachusetts Medical School, Worcester, MA USA; 2 Department of Human Genetics and Genomics, Laboratory of Evolutionary Genomics, Vavilov Institute of General Genetics of Russian Academy of Science, Moscow, Russia; 3 Department of Psychiatry and Department of Neuroscience, Friedman Brain Institute, Icahn School of Medicine at Mount Sinai, New York, NY USA; 4 Faculty of Biology, Center for Genetics and Genetic Technologies, Faculty of Bioengineering and Bioinformatics, Lomonosov Moscow State University, Moscow, Russia; 5 Program in Bioinformatics and Integrative Biology, University of Massachusetts Medical School, Worcester, MA USA; 6 Sirius University of Science and Technology, 1 Olympic Ave, 354340 Sochi, Russia

**Keywords:** Schizophrenia, Epigenetics in the nervous system

## Abstract

Both heritability and environment contribute to risk for schizophrenia. However, the molecular mechanisms of interactions between genetic and non-genetic factors remain unclear. Epigenetic regulation of neuronal genome may be a presumable mechanism in pathogenesis of schizophrenia. Here, we performed analysis of open chromatin landscape of gene promoters in prefrontal cortical (PFC) neurons from schizophrenic patients. We cataloged cell-type-based epigenetic signals of transcriptional start sites (TSS) marked by histone H3-K4 trimethylation (H3K4me3) across the genome in PFC from multiple schizophrenia subjects and age-matched control individuals. One of the top-ranked chromatin alterations was found in the major histocompatibility (MHC) locus on chromosome 6 highlighting the overlap between genetic and epigenetic risk factors in schizophrenia. The chromosome conformation capture (3C) analysis in human brain cells revealed the architecture of multipoint chromatin interactions between the schizophrenia-associated genetic and epigenetic polymorphic sites and distantly located *HLA-DRB5* and *BTNL2* genes. In addition, schizophrenia-specific chromatin modifications in neurons were particularly prominent for non-coding RNA genes, including an uncharacterized *LINC01115* gene and recently identified *BNRNA_052780*. Notably, protein-coding genes with altered epigenetic state in schizophrenia are enriched for oxidative stress and cell motility pathways. Our results imply the rare individual epigenetic alterations in brain neurons are involved in the pathogenesis of schizophrenia.

## Introduction

Schizophrenia (SZ) is a complex disorder with highly variable clinical manifestations of psychotic symptoms, such as auditory hallucinations, paranoid or bizarre delusions, disorganized thinking, and significant social dysfunction. Considerably higher concordance in monozygotic twins than in dizygotic twins or siblings indicates that there is a genetic component to this disease. The genetic methods, such as linkage in pedigrees and large scale genome-wide association studies (GWASs) have been employed to identify the genomic loci contributing to schizophrenia^1^. No robust evidence has been obtained showing the contribution of common genetic variations in the majority cases of SZ. Many studies have identified genomic loci putatively associated with SZ, among which the major histocompatibility complex (MHC) locus, which includes the cluster of human leukocyte antigen (HLA) genes, on chromosome 6, has emerged as the most robust signal in many GWAS studies, along with several other weaker signals on various chromosomes^2^. A recent direct search of rare variations and de novo mutations in protein-encoding gene regions in individuals with SZ revealed no significant overlap for the identified mutant genes in independent studies^3–7^. Collectively, few genetic markers have been discovered, which together explain a small proportion of the estimated genetic component. It is conceivable that many rare genetic variants with relatively modest or week effects may contribute to schizophrenia phenotype.

The human genome is decorated by numerous chemical modifications, jointly referred as “epigenetics”. They include direct modifications of DNA bases (for example, methylation of cytosines) and also changes of histones, which form a nucleosome—a protein complex DNA wraps around. Both of these epigenetic modifications are connected to gene expression^8,9^. For example, H3K4me3 marks promoters of active genes^10,11^. Interestingly, unlike genetic variants, epigenetic modifications are subject to environmental factors and change during lifetime. Therefore, epigenetics may at least partly explain the unexplained proportion of SZ genetic component.

Several studies have explored the epigenetic changes in SZ comparing to controls (CTRL), but their significance in schizophrenia neuropathology is still unclear^12–15^. Some studies investigated the epigenetics of the peripheral blood cells, but the brain epigenomic profiling is more relevant in the context of SZ. In particular, the prefrontal cortex (PFC) plays a major role in cognition and psychiatric diseases. However, brain epigenetic studies are complicated due to the heterogeneity of brain tissues, which contain many different cell types. While recently emerged single-cell epigenomic profiling is a very promising approach^16^, sorting and analysis of whole specific cell population allow us to investigate larger case-control cohorts. For, example RNA binding protein fox-1 homolog 3 (NeuN) can be used to select neuronal nuclei specifically and exclude non-neuronal brain cells from analysis^17–20^.

Using epigenomic profiling of H3K4me3, which is highly regulated in the developing and mature PFC^17^, we performed outlier analysis and explored epigenetic chromatin variations in cortical neurons specific for patients with SZ. We identified rare specific epigenetic variations in neurons of schizophrenia patients. The enrichment for rare up- or down open chromatin signals in schizophrenia were found for a set of novel non-coding RNA genes and genes essential in certain cellular pathways. We hypothesize that such rare epigenetic variants may affect regulation of gene expression and contribute to pathogenesis of SZ.

## Materials and methods

### H3K4me3 ChIP-seq data

We analyzed whole-genome ChIP-seq data obtained for chromatin regions marked by H3K4me3-specific antibodies in dorsolateral PFC neurons extracted from postmortem brain samples in a cohort of 16 unrelated patients with SZ and 16 age-matched CTRL individuals. For the accurate comparative analysis the identical experimental and bioinformatics protocols were used to generate ChIP-seq data for SZ and CTRL^17–20^ (Supplementary Table 1).

### Bioinformatic analysis

For ChIP-seq data analysis, we used ChIP-seq reads mapped to the human reference genome GRCh37 with Bowtie2^21^ using default parameters. We applied MACS 1.4.3^22^ to identify 29,547 loci of H3K4me3 signal enrichment in human prefrontal neuronal samples from a cohort that includes a subset of SZ individuals^23^ (with *P-*values of less than 1 × 10^–10^ against an input sample) and simultaneously generate coverage tracks for each sample. To account for unequal sequencing depth, we first generated a list of promoter regions for known genes from Ensembl v87 annotation (we collected transcriptional start sites (TSSs) for all transcripts, generated 4 kbp regions centered in TSSs and merged overlapping regions), for each sample tallied the total number of reads mapped to these regions, divided this tally by 10^7^, and then normalized the coverage at each base pair by the resulting number. Finally, for each sample we quantified the coverage in each of 29,547 regions as the sum of normalized coverages at each base pair of a particular region.

In order to detect regions enriched in H3K4me3 in samples from patients with SZ against all CTRLs, we selected upregulated peaks for which at least one individual with SZ had at least a 1.5-fold change compared to every of 16 CTRLs and with normalized coverage of at least 5. For downregulated peaks, we selected peaks for which at least one patient with SZ had at most a 0.5-fold change compared to every of 16 CTRLs and every CTRL have a normalized coverage of at least 5. In addition, we computed the nominal *P*-value for each peak individually as the probability of drawing the SZ coverage value for normal distributions with parameters estimated from all CTRL coverage values and required it to be less than 0.01. In both analyses, we also excluded peaks with low mapping quality reads (if 75% of reads in the peak had MAPQ < 10) and loci with low normalized coverage (<2.5). The stringent threshold of fold change compared to all CTRL individuals provides diminished positive rate while missing signals are expected (false negatives).

For both DAVID^24^ and ConsensusPathDB^25^ analyses, we selected genes with TSSs of protein-coding transcripts in close proximity (<2 kb) to one of the altered peaks. We set false discovery rate threshold at 0.05 for this analysis.

Intersections of genetic datasets for GWASs, exome data, and de novo mutations with epigenetic datasets for H3K4me3 variations in individuals with SZ were analyzed. For GWASs, we obtained raw *P-*values for all evaluated variants and generated genomic sets by selecting all variants with *P*-value < 5 × 10^–8^ and including 100-kb flanking regions. To evaluate significance of overlap, we used H3K4me3 peaks that overlapped with these genomic sets and the whole genetic variant set evaluated in the GWAS as a background. For exome (both case-control and *de novo* mutations) studies, we selected all SZ-altered peaks close (2 kb) to the TSSs of protein-coding genes reported in corresponding studies. We used all protein-coding genes as a background in this analysis. For CNVs, we used the whole genome as a background. For SZDB analysis, we also used all protein-coding genes as a background. We applied one-tailed Fisher’s exact test to evaluate statistical significance for overlap of up-, down- and all peaks for both SZ14 and SZ2 groups separately (six tests per each dataset) with false discovery rate <0.05.

### Chromosomal conformation capture at the HLA-DRB9 locus

Postmortem PFC brain tissues for three SZ cases and three CTRLs were obtained from the University of Maryland and pair-matched for age, sex, postmortem interval, and pH (Supplementary Table 2). In order to detect DNA looping interactions 3C libraries were prepared as previously described^26^. 3C primers (Supplementary Table 3) were designed based on the following criteria: (i) distance less than 200 bp from the HindIII restriction site; (ii) 30 bp in length; (iii) GC% of 40–45%; and (iv) melting temperature between 59 and 62 °C.

## Results

### Genomic regions showing outlier chromatin changes marked by H3K4me3 in SZ

We performed comparative analysis of datasets of H3K4me3 profiling in PFC neurons from patients with SZ (*N* = 16) and CTRL individuals (*N* = 16; Supplementary Table 1). Genome-wide correlation analysis demonstrated that H3K4me3 profiles of two unrelated patients with SZ (S10 and S11) were different (mean Pearson correlation coefficient, PCC = 0.89) from all other individuals but showed much higher similarity to each other (PCC = 0.97; Supplementary Figure 1). No particular clinical phenotypes or treatment specifically shared by these two subjects have been detected in available medical records. To exclude potential unknown confounding factors, we separately analyzed these two samples (SZ2 group) and another 14 individuals (SZ14 group).

**Fig. 1:**
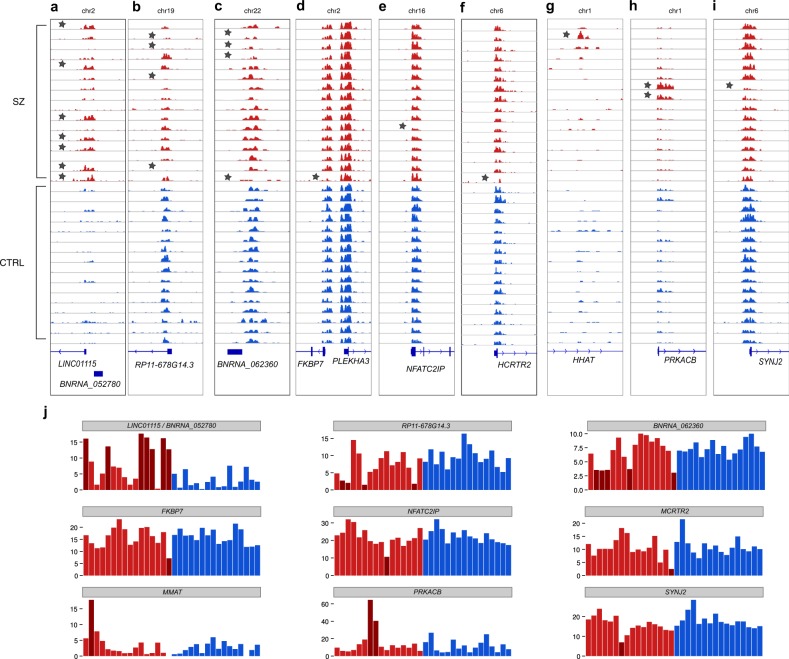
Examples of alterations in chromatin loci in patients with SZ. In SZ14 group: **a** the top most commonly upregulated loci for the ncRNA *LINC01115* and the novel *BNRNA_052780* gene (H3K4me3 peak genomic coordinates are chr2:862857–865643). **b** The top most commonly downregulated loci for the *RP11–678G14.3* gene (peak coordinates are chr19:21768973–21770226) and (**c**) the novel *BNRNA_062360* gene (peak at chr22:37720662–37721774). Examples of rare up and down peaks for (**d**) *FKBP7* (peak at chr2:179342628–179344328), (**e**) *NFATC2IP* (peak at chr16:28961457–28963616), (**f**) *HCRTR2* (peak at chr6:55038831–55041158), (**g**) the intron of *HHAT* in a single individual with schizophrenia (peak at chr1:210542908–210543238). Examples in SZ2 group: (**h**) *PRKACB* (peak at chr1:84629405–84633144), and (**i**) synaptojanin 2 (*SYNJ2*; peak at chr6:158401671–158404195). **j** Quantitative representations of peak sizes for SZ and CTRL groups for the above examples. Samples with SZ are colored in red; control individuals are colored in blue. SZ samples with significant H3K4me3 peaks size change are marked with a star in panels a–i and by dark read in panel j. *P*-values and fold changes for each SZ sample are presented in Supplementary Tables 4 and 5

To evaluate the roles of rare H3K4me3 variations, we searched for loci with significant individual alterations in patients with SZ with at least 1.5-fold changes in particular SZ individual compared to all control individuals (Fig. 1) to focus on the most robust individual epigenetic variants. For the SZ14 group we found 561 different genomic loci, with 227 upregulated loci and 335 downregulated loci in SZ (one locus was simultaneously upregulated in patient S8 and downregulated in patient S6; Supplementary Table 4, Supplementary Figure 2). In the SZ14 group, most of the upregulated or downregulated loci (89%) were observed in a single patient only, thereby representing individual-specific alterations. However, there were 56 loci (22 upregulated loci and 34 downregulated loci) with simultaneous alterations in at least two samples. For the SZ2 group, we found 1,265 loci (1,003 upregulated loci and 262 downregulated loci; Supplementary Table 5, Supplementary Figure 3) with 196 (15%) regions simultaneously altered in both samples.

**Fig. 2:**
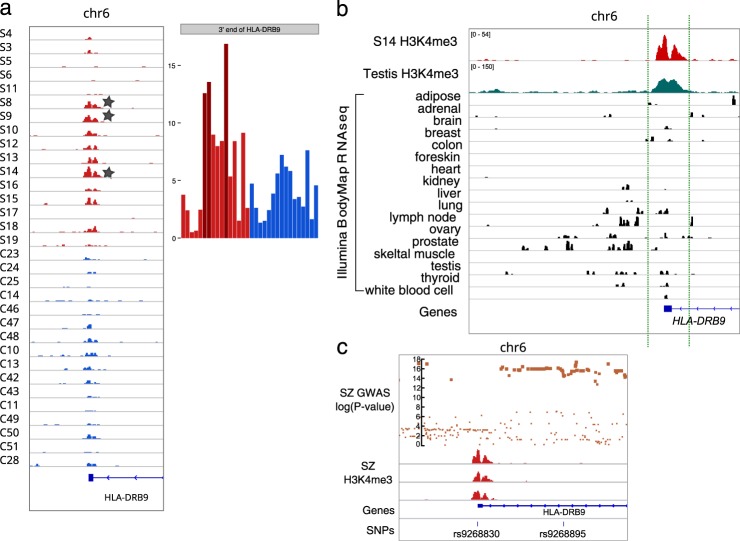
Analysis of the HLA-DRB9 locus. **a** The H3K4me3-marked open chromatin peak at the distal 3′-region of *HLA-DRB9* was upregulated in neurons from individuals with schizophrenia (indicated by a star); peak genomic coordinates are chr6:32427120–32428371. **b** The transcription activity(Illumina BodyMap 2.0 data) and chromatin state^62^ suggest common activity of this locus in the testis. **c** A cluster of significant variations from GWAS data for schizophrenia next to the peak (18). The log Y-scale represents reported nominal *P-*values for variants (36,989 cases and 113,075 controls). Bottom panel: the SNPs rs9268830 (inside the peak) and rs9268895 (the most significant SNP associated with schizophrenia in GWAS data) (19). SZ samples are marked with S prefix, control individuals are marked with C prefix. Red and blue colors indicate SZ and CTRL, respectively

A notable example in the SZ14 group was upregulation of the H3K4me3 peak (Fig. 1a, j) for individuals with SZ on chromosome 2 harboring TSS for two ncRNA genes, i.e., *LINC01115* and the novel unannotated previously gene *BNRNA_052780*, which is in a group of novel ncRNA genes that we have recently discovered^23^. Thus, this analysis revealed a single common neuronal marker shared by up to 50% of individuals with SZ, which formed a homogenous group in genome-wide correlation analysis of H3K4me3 profiles. In addition to the *LINC01115/BNRNA_052780* locus, the top upregulated locus was identified in the 3′ end of *HLA-DRB9* (Fig. 2a) altered in three patients with SZ. The most robust downregulated loci included a locus on chromosome 19 in close proximity to the ncRNA *RP11–678G14.3* **(**Fig. 1b**)** and on chromosome 22, with the predicted novel ncRNA gene, *BNRNA_062360* (Fig. 1c). Each of these two downregulated loci was altered in four individuals with SZ.

### The epigenetic roles of the HLA-locus and immunogenes in neurons

One of the two top H3K4me3 upregulated loci in SZ (the SZ peak) was identified in the 3′ end of the *HLA-DRB9* pseudogene located between the *HLA-DRA* and *HLA-DRB5* genes (Fig. 2a). This upregulation was found in at least three men with SZ (patients S8, S9, and S14), ages 50, 45, and 22 years old, respectively, and tended to be male-specific (Fisher’s exact test nominal *P-*value = 0.02). Moreover, this locus was strongly marked by H3K4me3 in the testis (Fig. 2b).

**Fig. 3:**
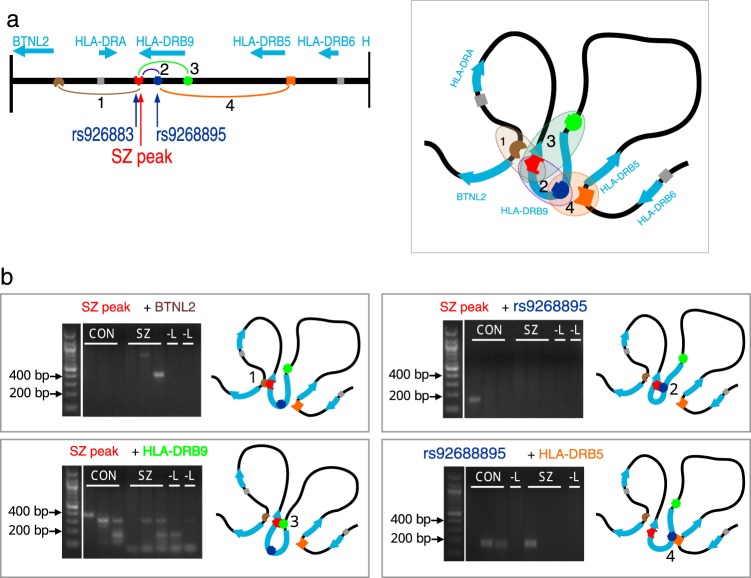
Putative looping interactions in the HLA-DRB9 locus. **a** Schematic representation of genes near the peak and putative looping interactions between SZ peak, SNP rs9268895, and nearby genes *BTNL2*, *HLA-DRB9*, and *HLA-DRB5*. **b** 3C experimental analysis of cells from postmortem cortical tissues revealed at least four looping interactions. SZ schizophrenia; CON control; -L no ligase

Interestingly, this peak was close to a cluster of single nucleotide polymorphisms (SNPs), which are strongly associated with SZ^27^ (Fig. 2c). Among these SNPs, rs9268895 has been previously reported to have the strongest association with SZ across the whole genome (under identifier rs114002140) with a nominal discovery *P*-value 8.28 × 10^–15^ ^28^. We found that another SNP (rs9268830) is located within the H3K4me3 SZ-peak, and its G allele showed strong linkage to the A allele of rs9268895 (*D*′ = 0.964, Supplementary Figure 4b). This H3K4me3 peak, located in the 3′ region of the *HLA-DRB9* gene, is far from any TSS for any known gene in this region (>14 kb). To elucidate the presumable chromatin interactions in this locus, we performed 3C-analysis in human brain samples. We anchored primers at the H3K4me3 peak, the GWAS significant SNP rs9268895, promoter of the distantly located *BTNL2* gene, and the TSS of the pseudogene *HLA-DRB9* to test for physical looping interactions with genes in the region in pair-matched postmortem PFC samples from individuals diagnosed with SZ and CTRLs (Fig. 3a, Supplementary Tables 2, 3). We detected four sequence-verified interactions: (1) between the SZ-peak and *BTNL2*; (2) between the SZ-peak and rs9268895; (3) between the SZ-peak and the *HLA-DRB9* TSS; and (4) between rs9268895 and *HLA-DRB5* (Fig. 3b). Each looping interaction was identified by sequencing in at least one of the tested individual brain specimens. Our data indicated that the H3K4me3-marked region in the pseudogene *HLA-DRB9* formed a physical loop with nearby genes *BTNL2* and *HLA-DRB5*.

In addition to the HLA locus, immune response gene regions are the most commonly genetic loci associated with SZ and other psychiatric illnesses in GWASs^29,30^. Here, we made interesting observation. We found that at least 651 (51%) of 1289 known immunogenes (Supplementary Note) showed the active H3K4me3-marked chromatin state in human prefrontal cortex neurons. Among these genes, 13 (2.0%) and 30 (4.6%) were dysregulated in the SZ14 and SZ2 groups, respectively (Supplementary Table 6).

These data suggested that immunogenes may have a broad and direct role in neuronal function or presumable epigenetic responses of neurons to inflammatory immune factors.

### Gene ontology and molecular pathways for epigenetically modified SZ loci

To further elucidate whether protein-coding genes with promoters marked by up- or downregulated loci in patients with SZ were enriched for any particular biological pathways or gene ontology terms^31^, we used DAVID^24^ and ConsensusPathDB^25^ web services for analysis of SZ-associated H3K4me3 loci.

**Fig. 4:**
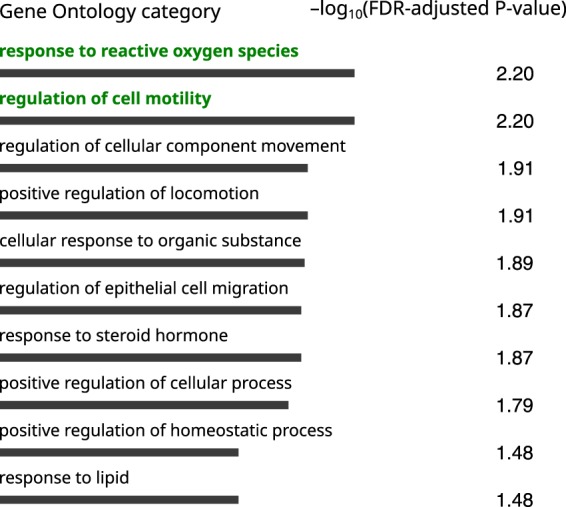
Top 10 enriched gene ontology terms analyzed with ConsensusPathDB for the SZ14 group. Terms with FDR-adjusted *P-*values of less than 0.01 are highlighted in green

In the SZ14 group, ConsensusPathDB analysis (Supplementary Table 7a) showed the strongest enrichment for genes involved in response to reactive oxygen species (false discovery rate (FDR)-adjusted *P* = 1.67 × 10^–5^) and regulation of cell motility (FDR-adjusted *P* = 3.12 × 10^–5^; Fig. 4a). For example, the gene for platelet-derived growth factor receptor, beta polypeptide (*PDGFRB*), which modulates the production of reactive oxygen species via NADPH oxidases^32^, is down-regulated in subject S13. The gene for one of multimerin 2 (*MMRN2*) isoforms, which regulates cell motility, is up-regulated in subject S12. This gene is located within a genomic region harboring 10q22–23 deletions, which are associated with behavioral abnormalities^33,34^. The pathways for response to reactive oxygen species and regulation of cell motility have been associated with schizophrenia: there is evidence of oxidative damage in SZ^35–38^ and cell motility is driven by actin filament dynamics^39^, which have been linked to *de novo* mutations in a recent study of patients with SZ^40^. DAVID analysis revealed no statistically significant enrichment. However, the top term was the phospholipase C (PLC) pathway, particularly hypocretin (orexin, *HCRT*) and hypocretin receptor 2 (*HCRTR2*), in a few individuals with SZ (Supplementary Figure 2, Supplementary Table 8; see Discussion).

In the SZ2 group, enrichment analysis demonstrated remarkable enrichment for the terms synapse (FDR-adjusted *P* = 2.1 × 10^–7^) and cell junction (FDR-adjusted *P* = 4 × 10^–7^) using DAVID (Supplementary Table 9); with ConsensusPathDB (Fig. 4b, Supplementary Figure 1l, Supplementary Table 7b), the top terms were nervous system development (FDR-adjusted *P* = 2.36 × 10^–6^) and cell differentiation (FDR-adjusted *P* = 9.38 × 10^–5^). Overall, these terms involved cadherins and their interacting partners, and some were markedly dysregulated in patients S10 and S11 (Supplementary Figure 3a–c). The difference in enrichment of gene pathways and ontology categories between SZ2 and SZ14 groups supports the global epigenetic reprogramming of genes for certain pathways in the prefrontal neurons in the two SZ2 samples. Although unknown confounding effects cannot yet be completely ruled out, these epigenetic differences may present a phenomenon of biological heterogeneity of the psychiatric disorder defined clinically under common SZ patterns.

Notably, we linked only 48% and 60% of SZ-altered loci to known protein-coding genes in the SZ14 and SZ2 groups, respectively, indicating that, correspondingly, 52% and 40% of regions were not included in the pathway analysis. Consistent with this, altered peaks were enriched for ncRNA genes in the SZ14 group (Bonferroni adjusted *P-*value = 0.007, Table 1). Thus, ncRNA genes may have a broader role than protein-coding genes in epigenetic alterations in SZ pathogenesis. Overall, we identified novel loci showing alterations in active chromatin for ncRNA genes, which may be involved in SZ. However, the functions of these alterations have not been evaluated yet.

**Table 1:** Enrichment of loci characterized by up and downregulated H3K4me3 peaks in schizophrenia in genomic regions harboring non-coding RNA genes or regions with no Ensembl annotated genes

H3K4me3 peak category	Total peak count	SZ14 up- and down-peaks (561)	
		Count	Bonferroni adjusted P-value
Ensembl annotated genes	20546	269	1.0
Protein-coding genes	16689	157	1.0
Non-coding genes	3857	112	0.00006
No Ensembl annotated genes (includes novel neuronal ncRNA genes^23^)	9000	192	1 × 10^–25^

### Integration of ChIP-seq data with high-throughput genetic studies

Next, we sought to test whether the H3K4me3 loci altered in neurons of individuals with SZ were enriched in the dataset of disease loci found in high-throughput genetic studies. The same genetic risk factors can contribute to different psychiatric diseases, particularly in SZ and affective disorders (e.g., bipolar disorder [BD] and major depressive disorder [MDD])^41^. Therefore, we downloaded the GWAS dataset reported for SZ (Schizophrenia Working Group, SWG set: 36,989 individuals with SZ and 113,075 CTRLs)^27^ and the GWAS data for five major psychiatric disorders (Cross-Disorder Group, GDG set: 33,332 cases and 27,888 controls)^41^. We investigated the overlap between H3K4me3 loci altered in SZ and the lists of SNPs (and the flanking 200-kb region) associated with psychiatric diseases. The SNP-associated loci were selected as sites with significant (nominal *P* < 5 × 10^–8^) and suggestive (nominal *P* < 1 × 10^–6^) thresholds (Supplementary Tables 10, 11). Comparatively, recent brain DNA methylation studies of SZ have shown weak^13^ or no^42^ direct overlap with the same SWG data. We also found a relatively modest or no sign of overlap for the ChIP-seq and GWAS signals for psychiatric illnesses (Supplementary Tables 10 and 11**)**. We found nominally significant overlap of epigenetic up- or downregulated H3K4me3 loci and loci harboring SNPs with suggestive associations (GWAS *P* < 1 × 10^–6^) with major depression disorder (MDD) in the GDG set for the SZ14 (*P* = 0.04) groups (Supplementary Table 11). In total, no significant enrichment was found for a set of epigenetic SZ loci and a set of multiple genetic loci putatively associated in GWASs with schizophrenia, autism spectrum disorder, attention deficit-hyperactivity disorders, or diabetes used as a control dataset (Supplementary Table 11) for the SZ14 group. We also found that, on a global level, the list of SZ-associated H3K4me3 loci was not significantly enriched by copy number variation (CNV) regions or by genes bearing rare de novo or inherited mutations found in SZ ^3,5,6,40,43^.

However, we found some specific genes showing overlap between our set of genes with epigenetic changes and a set genes for genetic risk for SZ and other psychiatric disorders (Supplementary Table 10). For example, the genes encoding L-type voltage-gated calcium channel subunits and dipeptidase 2 (*DPEP2*) were identified in many GWASs or in both GWASs and exome sequencing studies of SZ data **(**Supplementary Figure 2c). Interestingly, among the top seven up- and down-regulated H3K4me3 loci listed in the SZ14 group, at least two loci (*HLA* and *TRAF3IP2*) were strongly associated with SZ in SWG data, and a third locus harboring the ncRNA genes was assigned to 22q13.1, the chromosomal locus showing genetic association with SZ and abnormal behavior-associated 22q13 deletion syndrome^44^.

While globally the overlap with genetic data was not significant, when we used 2,706 genes from the SZDB database^45^, which includes not only GWAS data but also genetic linkage, gene expression, protein interaction, and convergent function genomics data, we found a nominally significant overlap with up-regulated peaks in the SZ14 group (nominal *P* = 0.007) (Supplementary Table 12). The overlap between differentially expressed genes from SZDB and epigenetically modified genes in SZ from the SZ14 group included the *LINC01115* ncRNA gene. The observed overlap supported the role of the identified loci and genes in SZ etiology.

## Discussion

In this study, we performed an outlier analysis of high-resolution mapping of the open chromatin mark, H3K4me3, which is primarily associated with active gene promoters, in PFC neurons of patients with SZ and CTRLs.

We identified multiple loci with up- and downregulated open chromatin in PFC neurons of patients with SZ versus CTRL individuals. For example, a locus in the promoter of the ncRNA *LINC01115* showed elevated H3K4me3 in half of our small set of schizophrenic patients. According to SZDB^45^, *LINC01115* RNA transcript was also reported to be upregulated in the PFC of patients with SZ in one study^46^. The function of *LINC01115* is poorly characterized. This gene is located ~100 kb upstream of *TMEM18*, which is well-known to associate with obesity^47^. A SNP in *LINC01115* has been reported to epistatically interact with *TMEM18*^48^. A methylation state of the CpG region located in *LINC01115* is linked to fat mass and fat free mass^49^. These reports suggest that the *LINC01115* gene/locus may regulate *TMEM18* expression. Body composition per se is altered in SZ^50^. Most interestingly, *TMEM18* modulates the migration of neuronal precursor cells and neural stem cells^51^. Notably, abnormal migration of neuronal progenitor cells has was also been reported for schizophrenia subjects^52^. Therefore, our analysis revealed a single putative neuronal marker for the gene, which may be involved in neural cell migration, shared by up to 50% of schizophrenic individuals with SZ in our sample set. Additional studies are needed to confirm these findings in a larger cohort.

Using gene ontology analysis, we found that the groups of genes with up- and downregulation of H3K4me3 loci were statistically significantly enriched with genes for oxidative response and regulation of cell motility in the SZ14 group. In addition, using DAVID, we observed the enrichment for the plasma membrane genes and cell motility in the top categories for the SZ14 group; however, these results were not statistically significant. Remarkably, regulation of phospholipase activity was one of the top pathways enriched in the downregulated loci for SZ. Phosphoinositide-phospholipase C-β1 (PLC-β1) is abnormally expressed in SZ^53,54^ and knockout of PLC-β1 in mice results in SZ-like phenotypes^55^. Within this pathway, receptor 1 of sphingosine 1-phosphate (S1PR1) is involved in activation of the PLC pathway^56^, and activation of HCRT and HCRTR2 increases PLC activity^57^. We found these genes downregulated in some patients with SZ.

Two patients with SZ in our dataset showed global chromatin alterations, particularly for synaptic genes, that were distant from those in other individuals with SZ. These data were consistent with recent independent observations of differential DNA methylation for some patients with SZ, which was very distant from others in SZ cohort groups^58^ and with observation that a subset of schizophrenia subjects form a separate group based on global alteration of transcriptome^59^. The segregation of SZ2 subjects from SZ14 group may be explained by distant etiology or symptomatology of SZ subtypes. Alternatively, it might reflect the drug effect that robustly changed the epigenomic state in SZ2 subjects^60^. To address this we showed no mix-up for the SZ2 samples that belong two independent subjects and that H3 K4me3 signal alterations overlap with genes showing change in expression under pharmacological induction and treatment of schizophrenia-like symptoms in primate-model (Supplementary Note, Supplementary Table 13) ^61^.

Our data reveal no overlap between epigenetically modified genes and genes bearing reported de novo mutations associated with schizophrenia^38^. Additional studies involving large cohorts of individuals with SZ need to be performed in order to estimate the total contributions of de novo mutations in H3K4me3-marked regions at risk for SZ. In total, our data demonstrated no global overlap between the loci showing H3K4me3 up- and down-regulation in schizophrenic subjects and schizophrenia-associated genetic loci implied in GWAS studies.

However, our results revealed a set of the genes showing both neuronal chromatin alterations and genetic associations with SZ. Notably, the region within the HLA gene cluster in the MHC locus on chromosome 6, which is the strongest and most common genetic risk factor for SZ^27^, also appeared to be a top epigenetically modified locus in SZ in this study. Furthermore, we demonstrated a potential physical looping interaction between identified region and nearby genes (*HLA-DRB5* and *BTNL2*), which suggests a role of novel promoter located in *HLA-DRB9* pseudogene or for non-promoter effect of this H3K4me3 signal in cortical neurons. However, if and how these DNA interactions are associated with SZ have yet to be studied.

Overall, in this study we demonstrated, that most of the epigenetic variations found specifically in individuals with SZ were rare and enriched at loci harboring non-coding genes. Interestingly, that the top H3K4me3 marked loci are also reported in top of genetic loci associated with schizophrenia (HLA locus on chromosome 6 and *TRAF3IP2* locus on chromosome 22) suggesting a potential causative role for such epigenetic changes. Given that many epigenetic changes are rare or private in the schizophrenia subjects they may represent random epigenetic variations that can be termed epi-mutations. We can hypothesize that such rare epi-mutations occurring in neurons could contribute to risk for schizophrenia. On the other hand, given the partial overlap for the global epigenetic alterations shared by some subjects, the subset of epigenetic signals can be a result of certain conditions associated with schizophrenia pathogenesis or environment, e.g. specific changes induced by drug therapy affecting neurotransmitter signaling. Finally, the study presents the list of novel ncRNA genes with epigenetic changes in schizophrenia, providing the new insight for further study of schizophrenia mechanisms.

## Supplementary information

Supplementary Data

Supplementary Tables
