# Supplementary material for: Chromatin profiling of cortical neurons identifies individual epigenetic signatures in schizophrenia
Source: Transl Psychiatry. 2019 Oct 17;9:256. doi: 10.1038/s41398-019-0596-1 (PMC6797775; doi:10.1038/s41398-019-0596-1)
Supplement: Supplementary file 1 — Supplementary Data [file 41398_2019_596_MOESM1_ESM.docx]

#

**Table of Contents**

[Supplementary figures 2](#__RefHeading___Toc542_3235586194)

[*Supplementary* Figure 1 2](#__RefHeading___Toc544_3235586194)

[*Supplementary* Figure 2 3](#__RefHeading___Toc546_3235586194)

[*Supplementary* Figure 3 9](#__RefHeading___Toc548_3235586194)

[*Supplementary* Figure 4 11](#__RefHeading___Toc550_3235586194)

[Supplementary tables 12](#__RefHeading___Toc554_3235586194)

[*Supplementary* Table 1 12](#__RefHeading___Toc556_3235586194)

[*Supplementary* Table 2 13](#__RefHeading___Toc558_3235586194)

[*Supplementary* Table 3 13](#__RefHeading___Toc560_3235586194)

[*Supplementary* Table 4 13](#__RefHeading___Toc562_3235586194)

[*Supplementary* Table 5 13](#__RefHeading___Toc564_3235586194)

[*Supplementary* Table 6 13](#__RefHeading___Toc566_3235586194)

[*Supplementary* Table 7 13](#__RefHeading___Toc568_3235586194)

[*Supplementary* Table 8 14](#__RefHeading___Toc570_3235586194)

[*Supplementary* Table 9 14](#__RefHeading___Toc572_3235586194)

[*Supplementary* Table 10 15](#__RefHeading___Toc574_3235586194)

[*Supplementary* Table 11 15](#__RefHeading___Toc576_3235586194)

[*Supplementary* Table 12 16](#__RefHeading___Toc578_3235586194)

[*Supplementary* Table 13 16](#__RefHeading___Toc580_3235586194)

[Supplementary Note 17](#__RefHeading___Toc582_3235586194)

[Kinship estimation 17](#__RefHeading___Toc1835_3804941228)

[Immunogenes list 17](#__RefHeading___Toc584_3235586194)

[Overlap of epigenetic changes in SZ2 group with drug-induced epigenetic alterations 17](#__RefHeading___Toc586_3235586194)

[References 18](#__RefHeading___Toc588_3235586194)

## Supplementary figures

In all figures showing H3K4me3 peaks subjects with schizophrenia (SZ) are designated in red and prefixed with S, while control individuals are in blue (CTRL) and prefixed with C.

### ***Supplementary*** Figure 1

**Genome-wide correlation analysis of CTRL and SZ samples. (a)** Samples S10 and S11 differ from other samples, but are similar to each other as indicated by genome-wide Pearson correlation. (b) Nucleotide counts for three SNPs in S10 and S11 extracted directly from ChIP-seq reads. The reads show different nucelotides in SNP
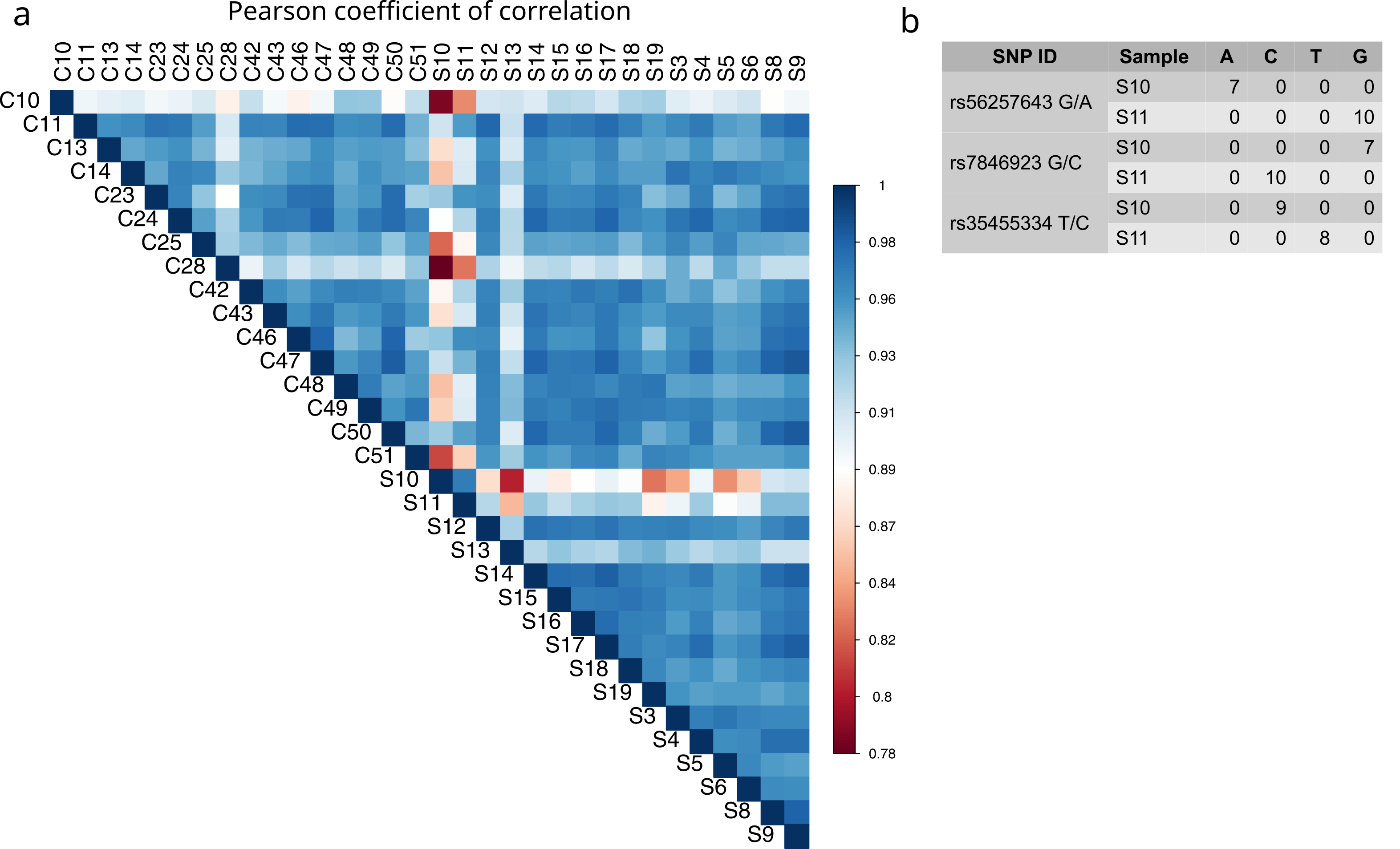
 homozygous state in S10 and S11 demonstrating no contamination between these samples.

### ***Supplementary*** Figure 2

**The genes with up-or down-regulated H3K4me3 marks in SZ14 cohort.** (**a**) Examples of peaks from top Gene Ontology terms in SZ14 group:*S1PR1* (peak genomic coordinates are chr1:101701221-101703383), *PDGFRB* (chr5:149533706-149535910), *MMRN2* (chr10:88727857-88729112). (**b**) Down-regulation of H3K4me3 for promoter of immunogene *CXCL5* in schizophrenic subject (peak coordinates are chr4:74863784-74864664).(**c**) Schizophrenia-associated genes found in genetic studies that show also the alterations in H3K4me3-enrichment in schizophrenic individuals: *DPEP2* (chr16:68027308-68027666), *SPNS1* (chr16:28985105-28987285), *CMIP* (chr16:81678319-81679741). (**d**) The *TRAF3IP2*, with altered H3K4me3 peak (chr6:111887888-111888561) in three schizophrenic subjects, is located in a locus strongly associated with increased genetic risk for schizophrenia (GWAS studies, max P-value = 1e-6). (**e**) Down-regulation of H3K4me3 for hypocretin (oxerin) neuropeptide precursor (*HCRT*; peak coordinates are chr17:40335491-40337099 and hypocretin (oxerin) receptor 2 (*HCRT2*; peak coordinates are chr6:55038831-55041158). (**f**) The orexin precursor and receptors interaction network [1]. (**g**) Control genomic region chr20:30101734-30103756 showing stable H3K4me3 pattern for gene for intramembrane protease (*HM13* or *SPP/IMPAS1*), but variability for the imprinted retrogene located in intronic region of *HM13* (chr20:30134374-30136203).


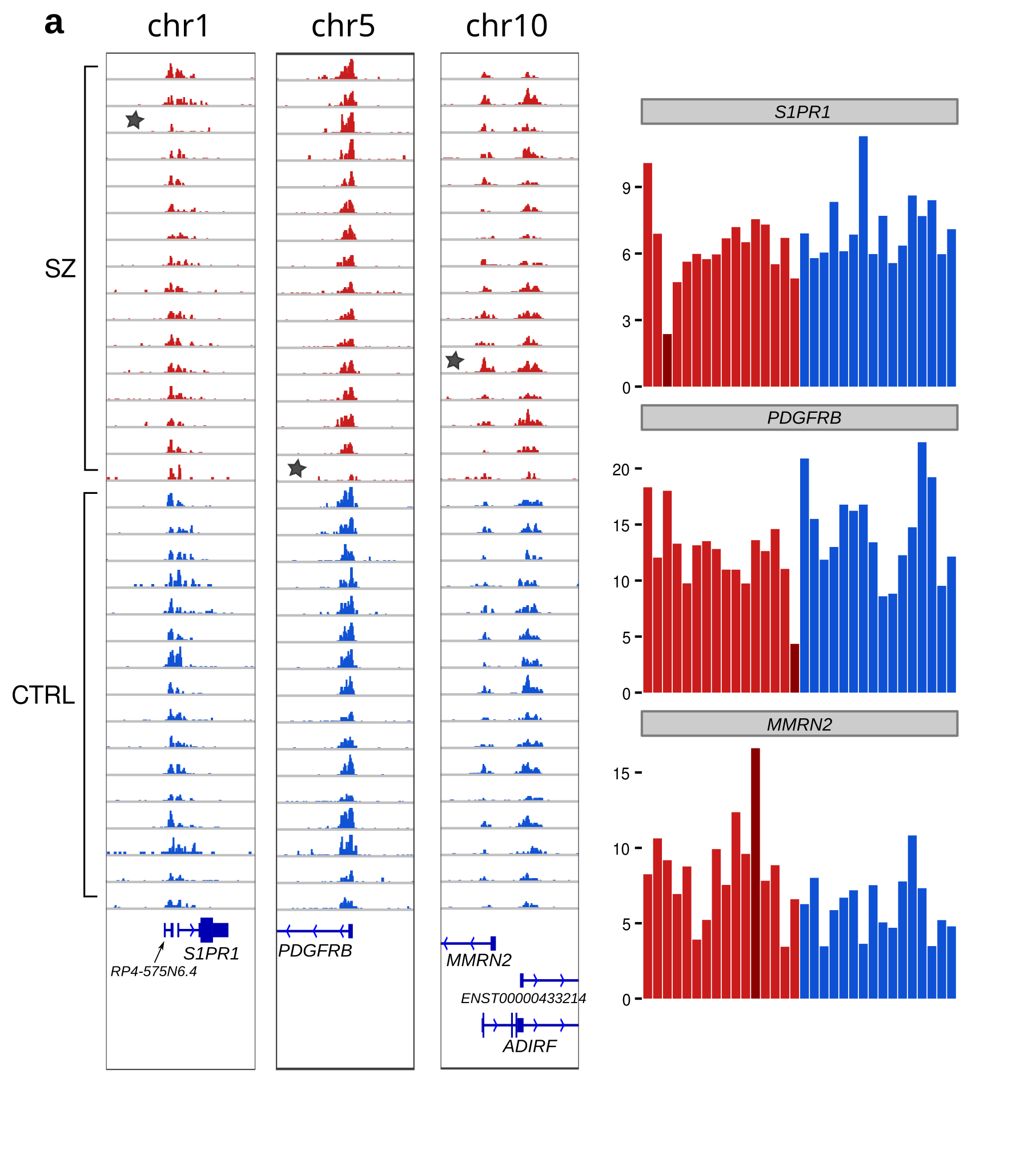

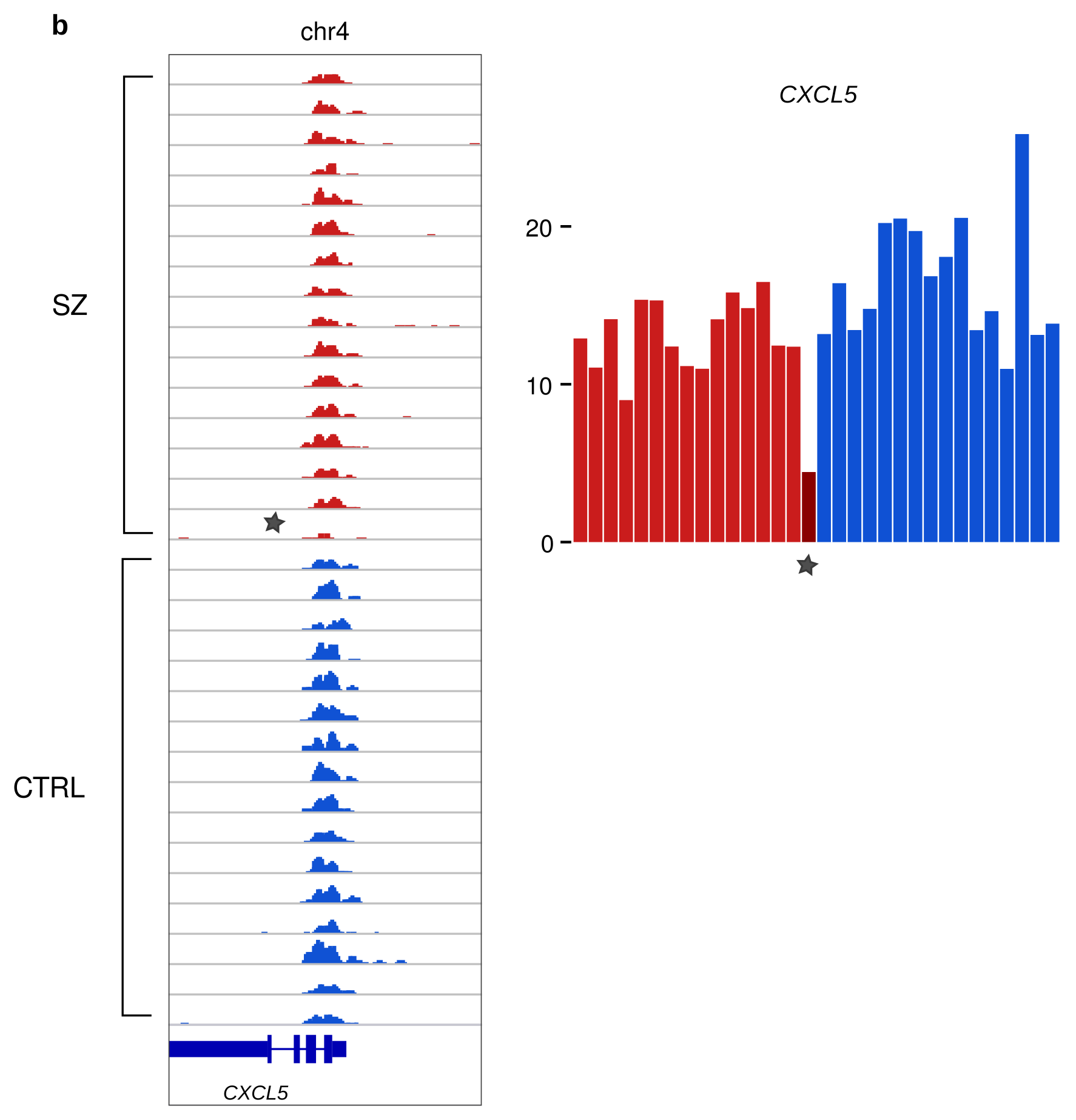

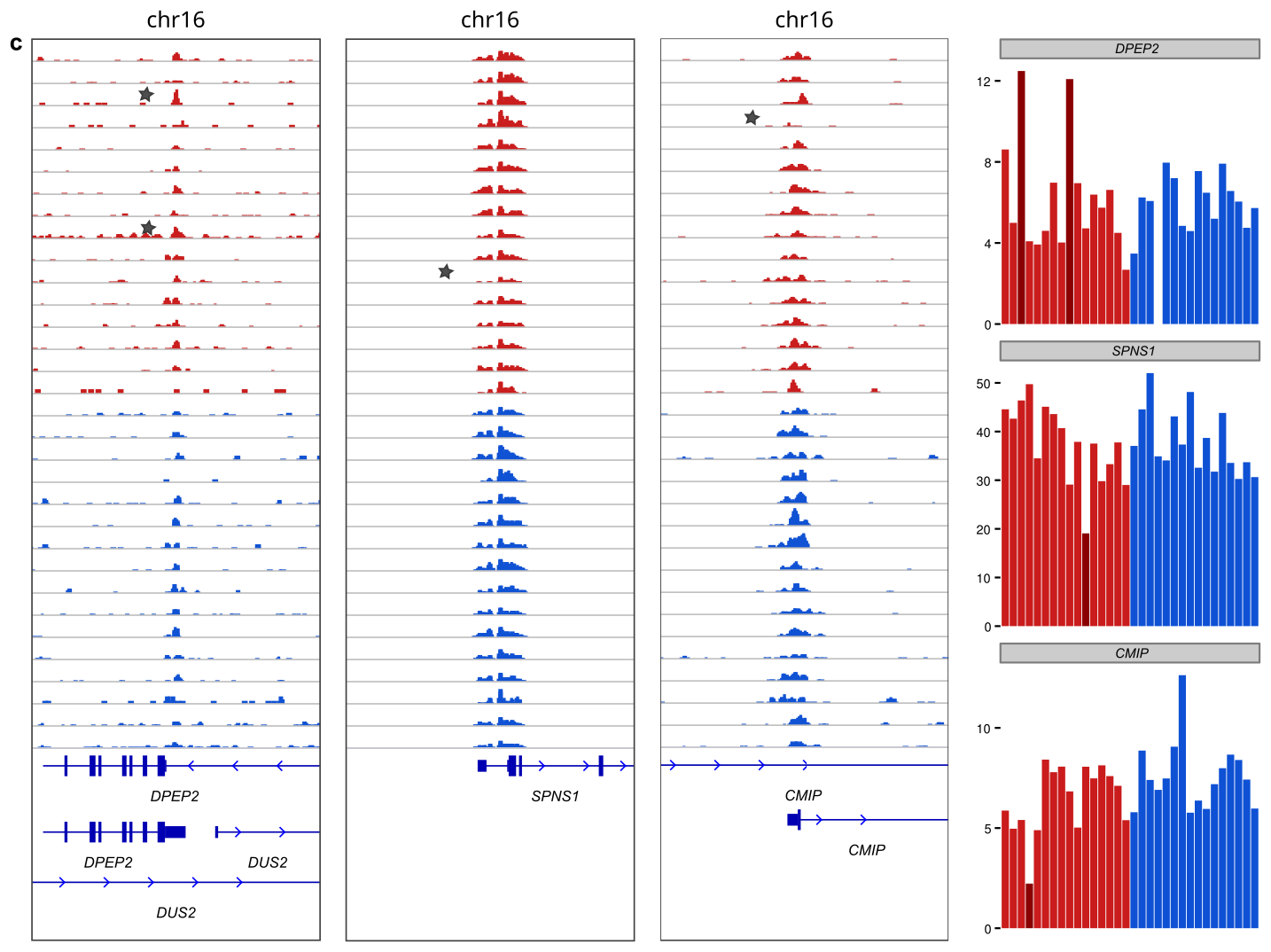

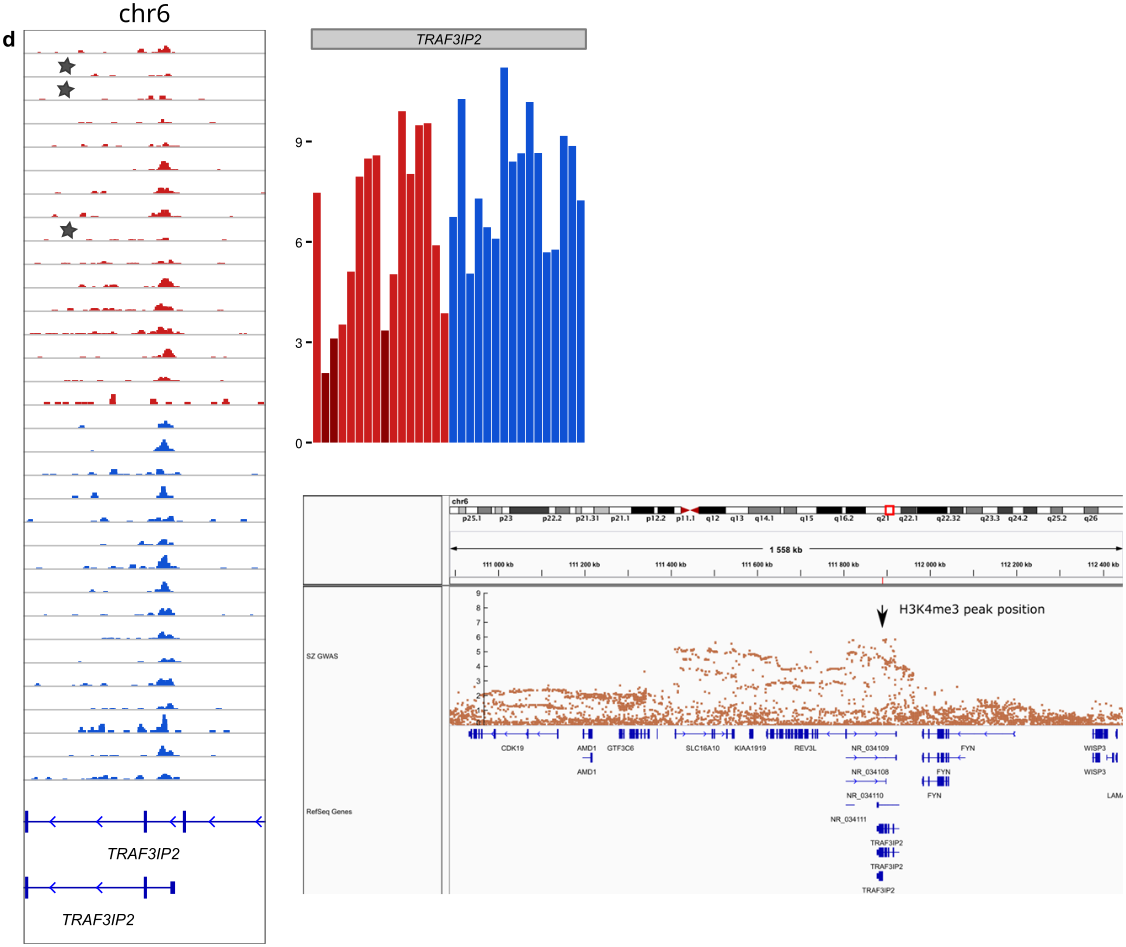

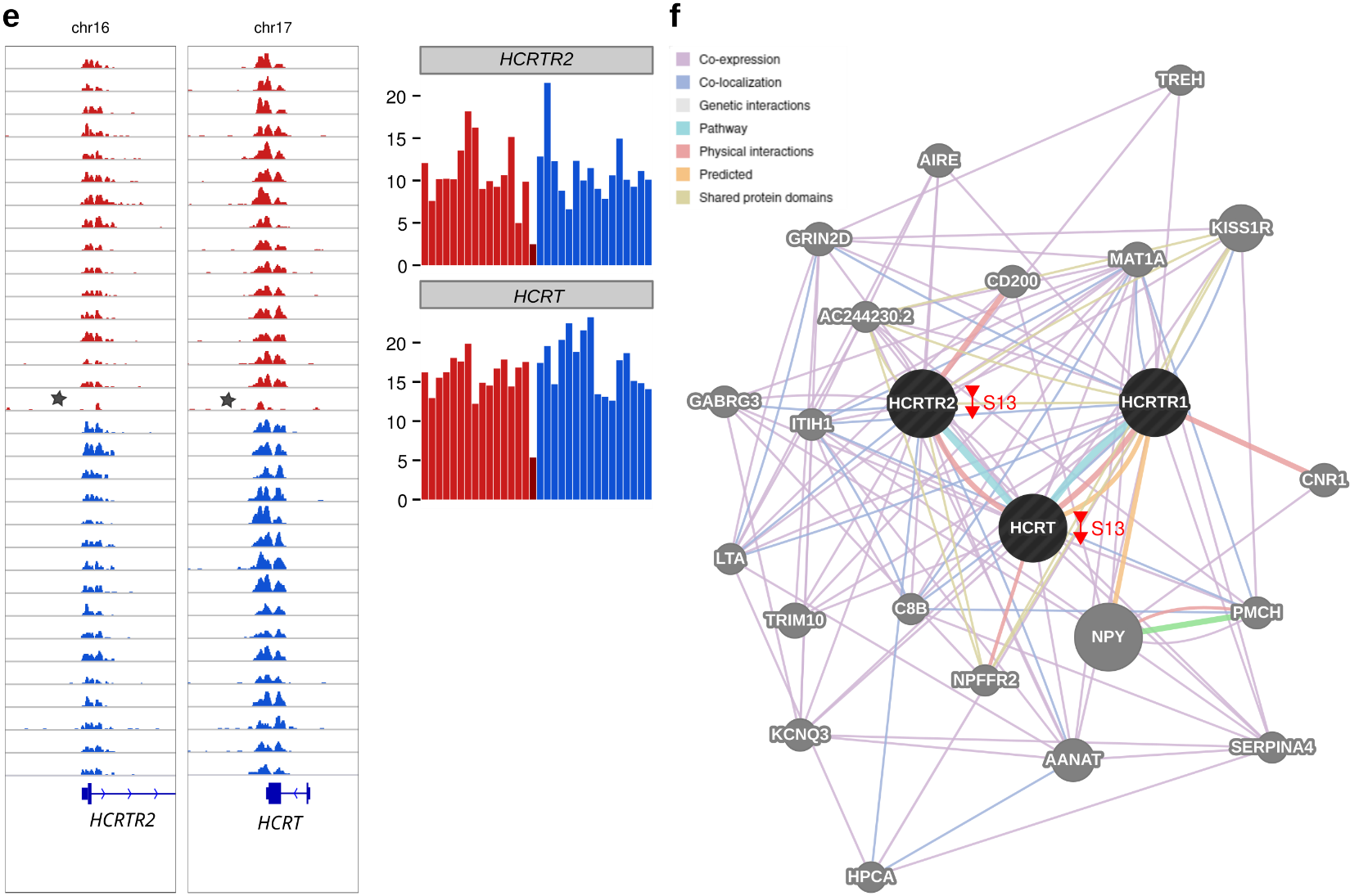

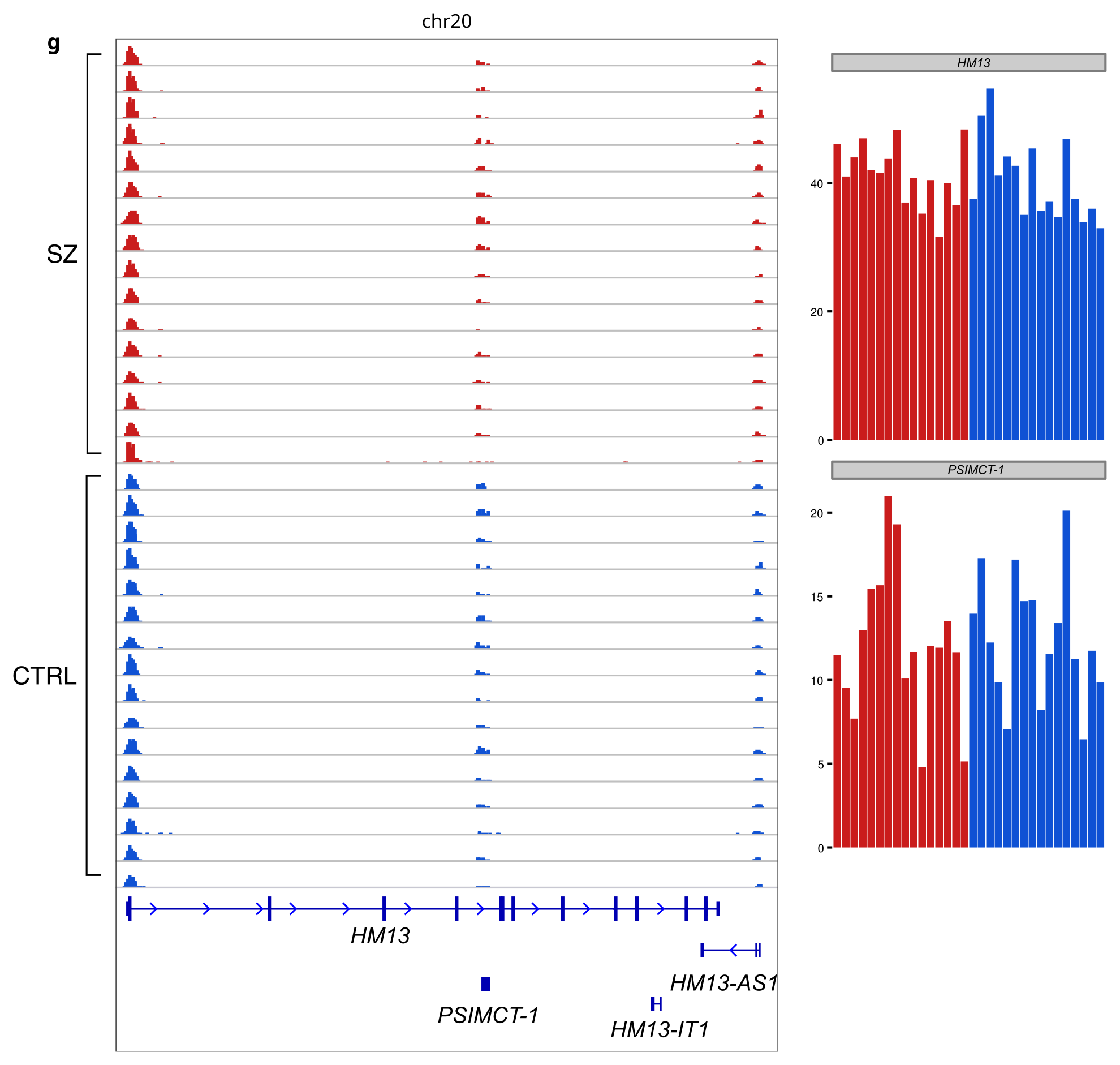


### ***Supplementary*** Figure 3

**The genes with up- or down-regulated H3K4me3 marks in SZ2 group.** (**a**) Alterations of cadherins and their interacting partners in subjects S10 and S11: *CDH10* (H3K4me3 peak coordinates are chr5:24640191-24646321), *CDH9* (chr5:27035254-27039186), *CDH18* (chr4:138452225-138454134), *PIK3R1* (chr5:67583534-67590744), *CTNNA2* (chr2:79412188-79414370), *CTNND1* (chr11:57528660-57533819). (**b**) Alterations of genes involved in synaptic pathways. (**c**) Schematic representation of neurotransmitter signaling and synaptic pathways [2] with genes showing H3K4me3 peak alterations in schizophrenic patients: *DLGAP1* (chr18:3870101-3875254), *PRKACB* (chr1:84629405-84633144), *AKT1* (chr14:105259979-105263023), *SYNE1* (chr6:152790307-152794104). The proteins for genes with up- or down-regulated H3K4me3 peaks in schizophrenia are marked by red stars.


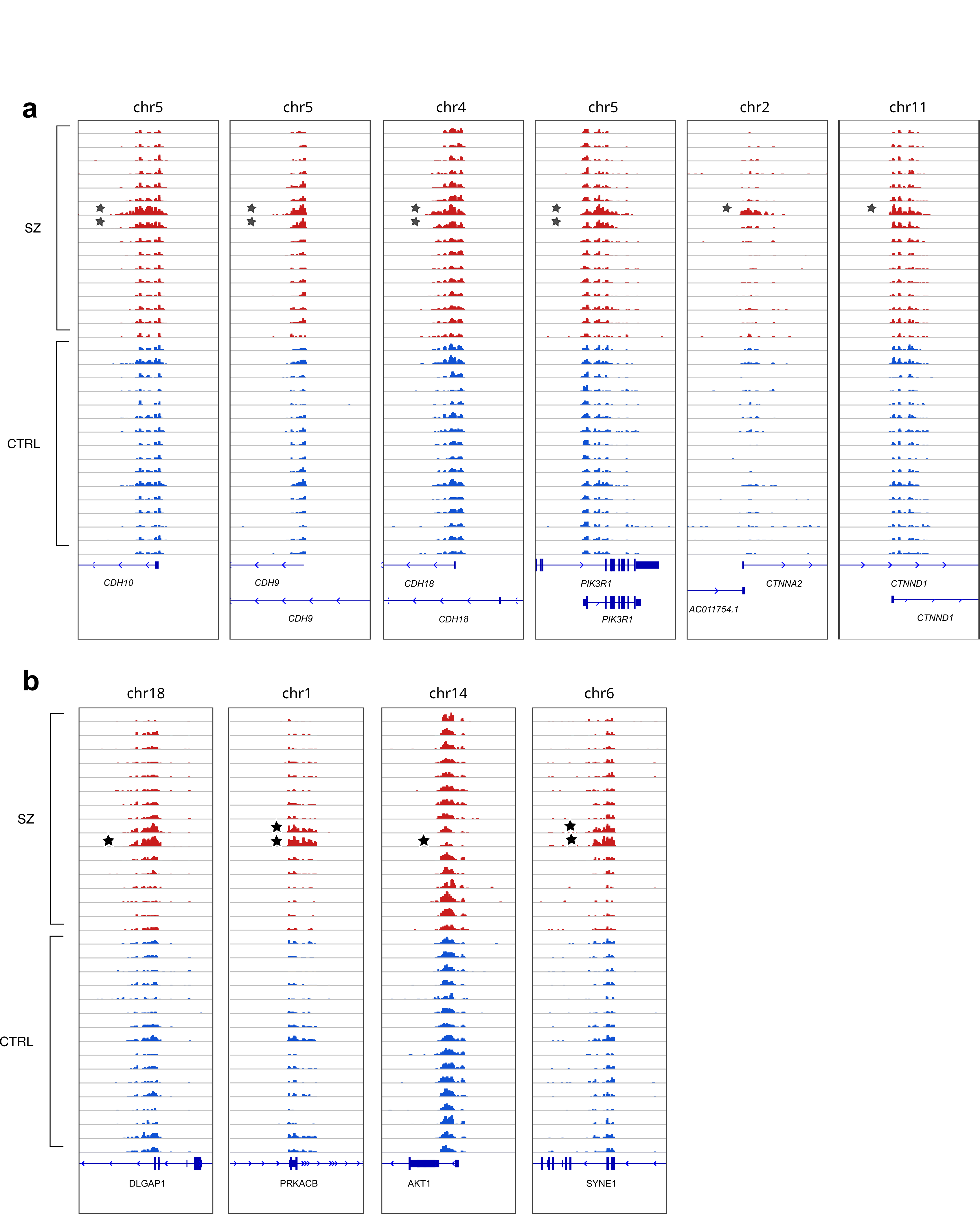


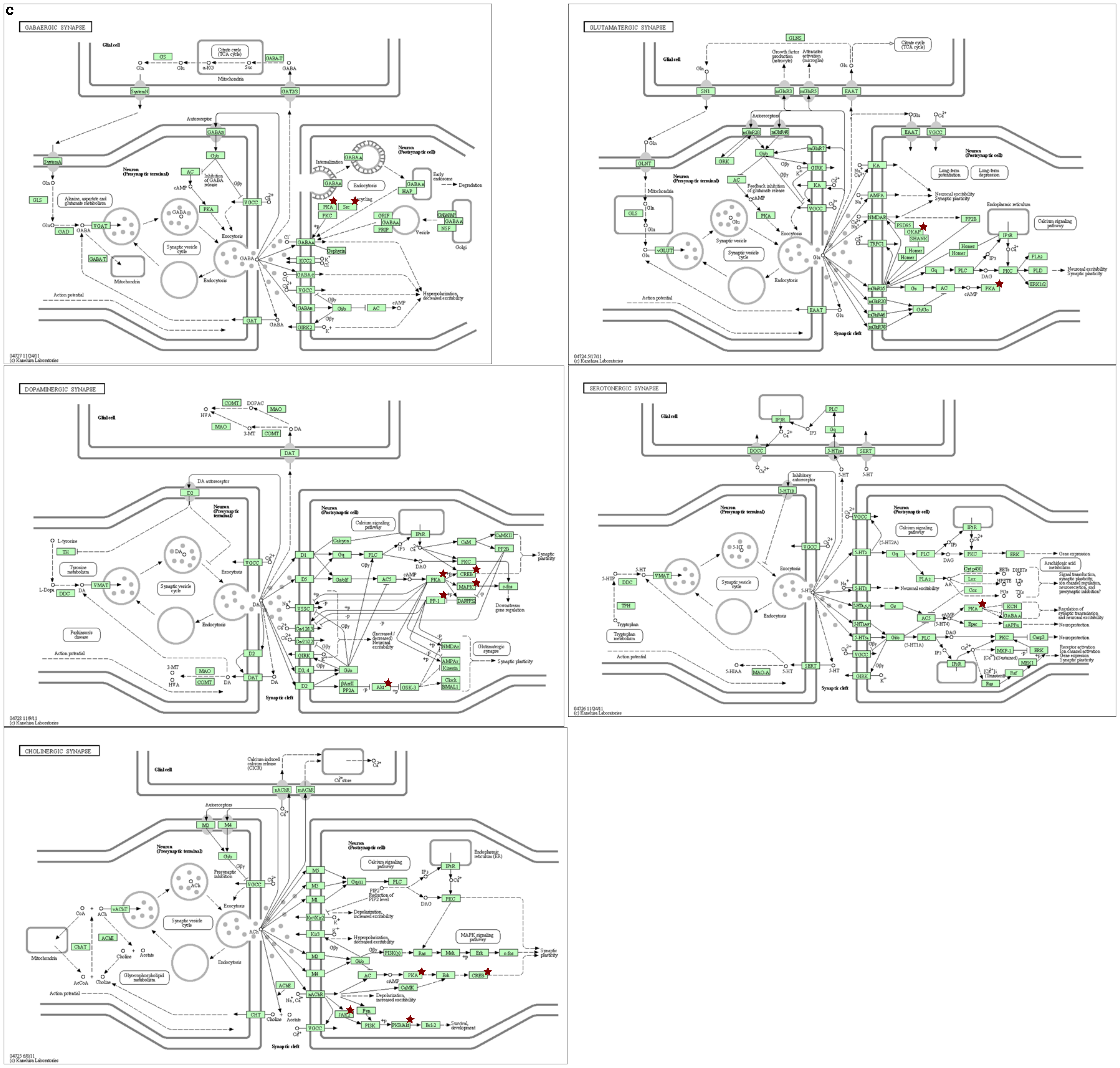


### ***Supplementary*** Figure 4

**Up-regulated *HLA-DRB9* H3K4me3 peak in schizophrenia.** (**a**) Genetic polymorphism found in chromatin interacting regions and genotyped in the cortical specimens demonstrated no apparent allele association with up-regulation of H3K4me3 peak (genomic coordinates of the peak are chr6:32427120-32428371). (**b**) The genotypes for 2504 unrelated human individuals from 1000 Genomes project phase 3 release. The close genetic linkage was found between rs9268895 located within the H3K4me3-peak and rs9268830 strongly associated with schizophrenia in GWAS studies.

##
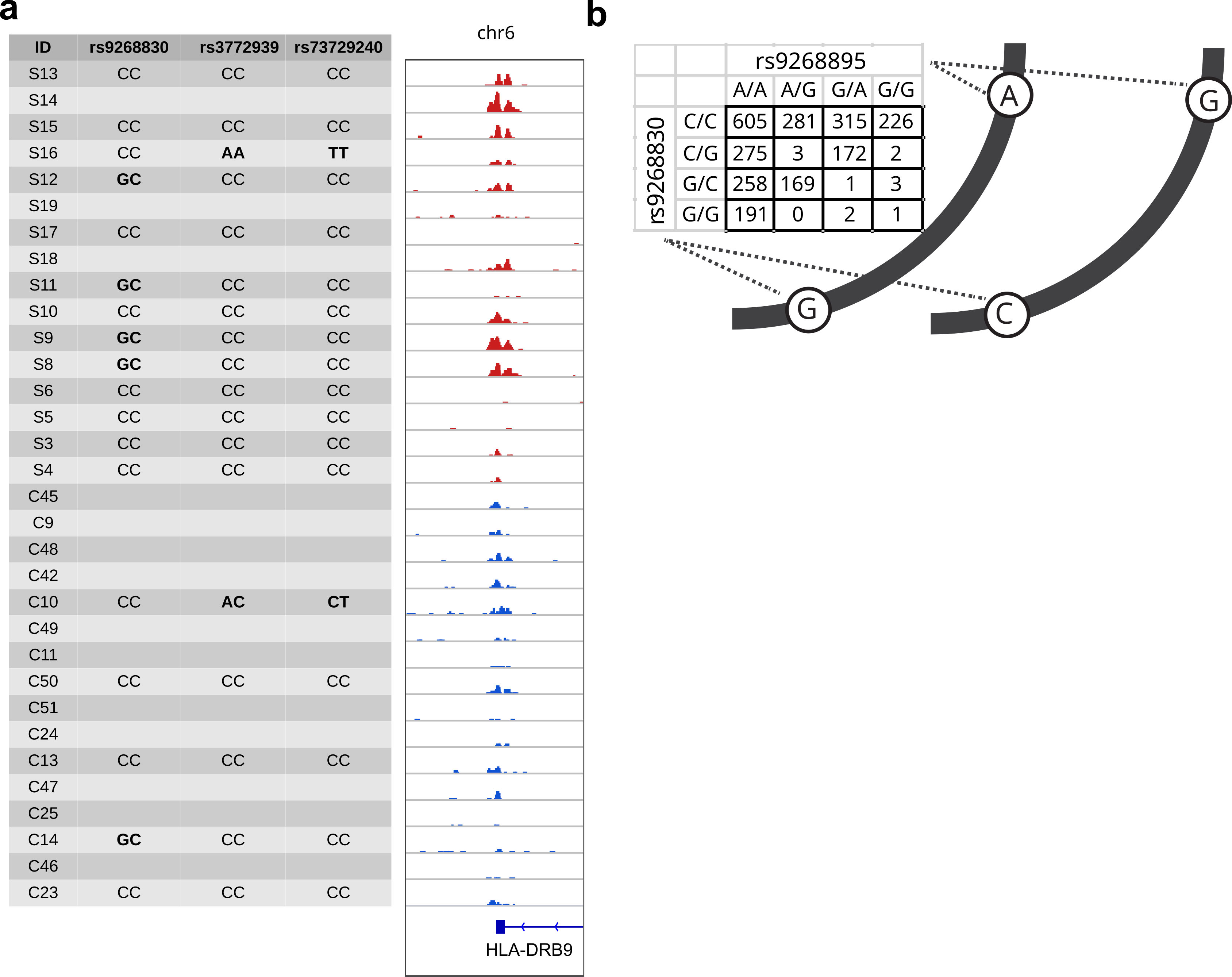
Supplementary tables

*Provided in a separate Excel (.xlsx) file.*

### ***Supplementary*** Table 1

**Sample description.**

Phenotype are denoted SZ (schizophrenia) and CTRL (no psychiatric diseases).

### ***Supplementary*** Table 2

**Primers used for the HLA-DRB9 3C experiment.**

### ***Supplementary*** Table 3

***List of immunogenes with altered H3K4me3 peaks in schizophrenic individuals.***

### ***Supplementary*** Table 4

**Genomic loci with altered H3K4me3 peaks in at least one individual of SZ14 group.**

The columns are:

- Locus – genomic coordinates of the locus
- Sample_count – how many samples have dys-regulation in this locus
- Direction – UP- or DOWN-regulation
- Known genes (coding) – known genes, which have a protein-coding transcript with transcription start site within 2 kb of the locus
- Known genes (non-coding) – same as above, but for non-coding transcripts
- Novel RNA-seq Genes – same for novel genes, predicted using RNA-seq and ChIP-seq [3]
- Samples – description of alternation in each sample: list of affected samples along with fold change, P-value and fraction of low-quality reads within locus (mapQ < 10) for each sample.

### ***Supplementary*** Table 5

**Genomic loci with altered H3K4me3 peaks in at least one individual of SZ2 group.**

Columns are as in Supplementary Table 4.

### ***Supplementary*** Table 6

**Demographics of postmortem cohort used for the HLA-DRB9 3C experiment.**

### ***Supplementary*** Table 7

**Enriched Gene Ontology terms: ConsensusPathDB analysis.**

This table contains 2 sheets (ST7a, ST7b) with results of over-representation analysis for Gene Ontology terms within genes, dys-regulated in at least one SZ individual (preformed only for Gene Ontology terms of level 4 using ConsensusPathDB web-service):

- ST7a– for genes, dysregulated in group SZ14
- ST7b – for genes, dysregulated in group SZ2

Hits, significant under q-value < 0.01, are highlighted with green.

### ***Supplementary*** Table 8

**Enriched Gene Ontology terms: DAVID analysis in SZ14 group.**

This table contains 3 sheets (ST8a, ST8b, ST8c) with results of over-representation analysis for Gene Ontology terms within genes, dysregulated in at least one SZ individual for group **SZ14** (preformed using DAVID web-service):

Sheets are:

- ST8a – analysis for all peaks (up- and down-) combined
- ST8b – analysis only for up-peaks
- ST8c – analysis only for down-peaks

Columns are (the same in all three sheets):

- Category – category of term
- Term – term ID and name
- Bonferroni, Benjamini – corrected P-values
- FDR - false discovery rate
- # of Affected Genes – how many genes are dys-regulated for this term
- # of Samples – how many samples have at least one gene dys-regulated
- Samples & Genes UP – samples with up-regulated genes (and gene names)
- Samples & Genes DOWN – samples with down-regulated genes (and gene names)

### ***Supplementary*** Table 9

**Enriched Gene Ontology terms: DAVID analysis in SZ2 group.**

This table contains 3 sheets (ST9a, ST9b, ST9c) with results of over-representation analysis for Gene Ontology terms within genes, dysregulated in at least one SZ individual for group **SZ2** (preformed using DAVID web-service)

Sheets are:

- ST9a – analysis for all peaks (up- and down-) combined
- ST9b – analysis only for up-peaks
- ST9c – analysis only for down-peaks

**The columns are the same as in Supplementary Table 8.**

### ***Supplementary*** Table 10

**Overlap between genes with altered H3K4me3 peaks in schizophrenia and disease-associated loci from genetic studies.**

This table contains 2 sheets:

- ST10a – analysis for group SZ14
- ST10b – analysis for group SZ2

Columns are:

- Locus – coordinates of up- or down-regulated H4K4me3 peak
- Dataset – the study, which reports overlapping gene or genemic locus
- Protein-coding genes – protein coding genes, which are located in proximity to H3K4me3 peak
- Non-coding genes – non-coding genes, which are located in proximity to H3K4me3 peak
- Novel genes – newly found genes [4], located in proximity to H4K4me3 peak

### ***Supplementary*** Table 11

**Significance of overlap between genes with altered H3K4me3 peaks in schizophrenia and disease-associated loci from genetic studies.**

### ***Supplementary*** Table 12

**Overlap between genes with altered H3K4me3 peaks in schizophrenia and SZDB.**

This table contains 4 sheets:

- ST12a – analysis of up-peaks in group SZ14
- ST12b – analysis of down-peaks in group SZ14
- ST12c – analysis of up-peaks in group SZ2
- ST12d – analysis of down-peaks in group SZ2

The columns are (the same for all sheets):

- Gene – gene, reported in SZDB
- Entrez ID – ID for the gene
- SZDB lines of evidence – reasons for this gene to be in SZDB
- H3K4me3 peak – genomic coordinates of the peak in close proximity to the gene
- Affected subjects – SZ subjects with up- or down-regulations of this H3K4me3 peak

### ***Supplementary*** Table 13

**Overlap between genes with altered H3K4me3 peaks in SZ and genes, which expression is altered by phencyclidine and olazapine**

The columns are:

- Gene category: PCP_VS_VEH – genes, which expression changed after administration of phencyclidine compared to vehicle (only one up-regulated gene is reported in [5], it was excluded from this analysis), PCP_OLZ_VS_PCP – genes, which expression changed after co-administration of olazapine with phencyclidine (only up-regulated genes are reported in [5], it was excluded from analysis), OLZ_reversed_genes – genes, which expression was reversed by co-administration of olazapine;
- SZ subject – genes near H3K4me3 peaks altered for this particular subject are compared to genes, altered in non-human primates by phencyclidine and olazapine;
- Peak direction – only up- or down-peaks are considered in this analysis;
- P-value – Fisher's exact test P-value for overlap of genes;
- Genes in overlap – list of genes found in overlap between epigenetically altered genes in SZ and genes with expression changes after phencyclidine and olazapine administration.

## Supplementary Note

## Kinship estimation

We estimated kinship coefficient between SZ subjects confirm no relatedness, including between S10 and S11 samples. Kinship coefficient are estimated using only common SNPs (minor allely frequency >5%) separated by at least 100 kbp with a maximum likelihood approach to account for unequal coverage [5].

## Immunogenes list

To compile a list of 1,289 known immunogenes, we collected genes listed in categories Cytokines, Cytokine Receptors, TCR Signaling Pathway, BCR Signaling Pathway, Natural Killer Cell Cytotoxicity, Antigen Processing and Presentation, Antimicrobials from the ImmPort database (<https://www.immport.org/shared/genelists>).

## Overlap of epigenetic changes in SZ2 group with drug-induced epigenetic alterations

Significant differences between groups SZ2 and SZ14 may be consequences of administered drug therapy. To address this issue we compared the epigenetic alterations found in our SZ cohorts with gene expression changes in non-human primates treated with phencyclidine, which creates SZ-like symptoms, followed with co-administration of olazapine, an anti-psychotic [61]. We observed that genes in close proximity to up-regulated H3K4me3 peaks are enriched with genes down-regulated by phencyclidine administration in both SZ2 subjects (Fisher’s exact test *P* = 0.0005 for subject S10, *P* = 0.0119 for subject S11; Supplementary Table 13), but not in any SZ14 subjects (*P* = 1). Furthermore, only in SZ2 group, but not in SZ14, up-regulated peaks are enriched with genes which expression is altered by consequent olazapine co-administration (*P* = 0.0001 for S10, *P* = 0.0133 for S11) , and specifically by genes, which reverse the changes induced by phencyclidine (*P* = 1×10-5 for S10, *P* = 0.003 for S11). At the same time, down-regulated peaks are not enriched with the same genes in neither SZ2 nor SZ14 subjects (*P*  > 0.05) after  olazapine adminis. Significant overlap of only up-regulated peaks in SZ2 group only suggests the connection of distinct epigenetic pattern in these group to medical treatment, for example, due to over-correction by some anti-psychotic similar to olanzapine. These initial observations highlight a need for accurate analysis accounting for SZ diversity and/or therapy effect in human brain epigenetic studies.

## References

1. Warde-Farley, D., Donaldson, S. L., Comes, O., Zuberi, K., Badrawi, R., Chao, P., Franz, M., Grouios, C., Kazi, F., Lopes, C. T., Maitland, A., Mostafavi, S., Montojo, J., Shao, Q., Wright, G., Bader, G. D., and Morris, Q. (2010) The GeneMANIA prediction server: Biological network integration for gene prioritization and predicting gene function. *Nucl. Acids Res.* **38**, W214–W220

2. Kanehisa, M., Goto, S., Sato, Y., Kawashima, M., Furumichi, M., and Tanabe, M. (2014) Data, information, knowledge and principle: Back to metabolism in KEGG. *Nucleic Acids Res.* **42**, D199–205

3. Gusev FE, Reshetov DA, Mitchell AC, Andreeva TV, Dincer A, Grigorenko AP et al. Epigenetic-genetic chromatin footprinting identifies novel and subject-specific genes active in prefrontal cortex neurons. The FASEB Journal 2019; 33: 8161–8173.

4. Pratto F, Brick K, Khil P, Smagulova F, Petukhova GV, Camerini-Otero RD. Recombination initiation maps of individual human genomes. Science 2014; 346: 1256442–1256442.

5. Lipatov M, Sanjeev K, Patro R, Veeramah KR. Maximum Likelihood Estimation of Biological Relatedness from Low Coverage Sequencing Data. bioRxiv 2015; : 023374.
